# A cluster of adenovirus type B55 infection in a neurosurgical inpatient department of a general hospital in Guangdong, China

**DOI:** 10.1111/irv.12457

**Published:** 2017-06-26

**Authors:** Lina Yi, LiRong Zou, Jing Lu, Min Kang, Yingchao Song, Juan Su, Xin Zhang, LiJun Liang, HanZhong Ni, Changwen Ke, Jie Wu

**Affiliations:** ^1^ Center for Disease Control and Prevention of Guangdong ProvinceGuangzhouChina; ^2^ Guangdong Provincial Institute of Public HealthGuangdong Provincial Centre for Disease Control and PreventionGuangzhouChina

**Keywords:** hospital infection, human adenovirus type 55, respiratory disease

## Abstract

**Background:**

Human adenovirus type 55 is a re‐emerging human respiratory pathogen that is associated with several respiratory infections outbreaks in military and school populations. In this study, we describe the first HAdV55‐associated hospital outbreak documented in Guangdong, China.

**Methods:**

Active surveillance was conducted in the involved neurosurgical inpatient department. All staff and patients in the involved neurosurgical department were surveyed for any symptoms of fever (≥38°C) and enlarged tonsils during the outbreak period. Throat swabs and demographic information were collected for all cases. For each specimen, assays for common respiratory viruses were performed using one‐step reverse transcription‐polymerase chain reaction. HAdV‐positive samples were inoculated onto Hep‐2 cells for isolation. Hexon genes, fiber genes, penton genes, and whole genomes were sequenced. A phylogenetic tree was constructed.

**Results and Conclusions:**

Forty‐three cases, including 24 laboratory‐confirmed cases and 19 possible cases, were identified. Nurses had the highest attack rate of infection, with a rate of 36.4%. The attack rate for doctors and inpatients was 20.0% and 16.7%, respectively. HAdV55 was the sole pathogen identified during this outbreak. The hexon, fiber, and penton genes from seven isolated HAdV55 stains were sequenced. All these genes showed 100% homology and fell into the HAdV55 [P14H11F14] cluster, indicating that HAdV55 was the single viral strain for the outbreak. While not conclusive, the epidemic investigation revealed that the outbreak was introduced by nurses from sources outside the hospital. It was likely that a transmission from staff to inpatients had occurred.

## 1 INTRODUCTION

Human adenovirus (HAdV) is a common pathogen among children and adults. Infections of HAdV can cause a variety of clinical diseases, ranging from asymptomatic and self‐limited conditions to pneumonia and even death.^1^ As of now, at least 68 HAdV genotypes have been identified and are classified into seven species (A‐G).^2^ Furthermore, viruses in species B, C, and E are more commonly associated with symptomatic respiratory infections.^1^, ^3^, ^4^, ^5^, ^6^, ^7^

Human adenovirus type 55 (HAdV55), a member of HAdV‐B family, was first identified from a military outbreak in Spain in 1969.^8^ This virus used to be recognized as HAdV‐B11a by partial characterization of its hexon and fiber epitopes.^9^, ^10^, ^11^ In 2006, Michael et al. revealed that HAdV55 was an emergent respiratory pathogen.^12^ It had evolved from a homologous recombination between HAdV‐B14 and HAdV‐B11 in its hexon gene, which conferred changes in viral serotype and immune activity. In recent decades, HAdV55 has been associated with several respiratory infections outbreaks.^9^, ^11^, ^13^, ^14^ Crowed communities, military training camps, and schools are common settings for HAdV infections. Here, we describe a HAdV55 outbreak occurred in a neurosurgical inpatient department of a Jiangmen hospital in Guangdong Province, China.

## 2 METHOD

### 2.1 Outbreak settings and investigation

On June 30, 2016, one local Center for Disease Control and Prevention of Guangdong was informed that several staff members, including nurses and doctors working in a general hospital, fell ill with symptoms of a fever and sore throat. The hospital is a district general hospital. The involved neurosurgical inpatient department is independently located on the eighth floor of the inpatient building. The department consists of a Neurosurgical Intensive Care Unit (NSICU) in the middle of the floor with 13 beds, and two normal neurosurgical units (A&B) on the right and left sides of the floor with 46 beds each. Units A and B each have seven doctors and 21 nurses/care‐workers. Both units have separate rooms for the doctors and the nurses/care‐workers. The NSICU had 24 nurses/care‐workers and was managed jointly by the doctors in units A and B. There was one acupuncturist responsible for the whole department. During the outbreak, the department had 90 inpatients, of which 33 were in unit A, 46 in unit B, and 11 in the NSICU.

By reviewing patients' medical records, we identified the first case and determined the investigation period began on June 11, 10 days before the onset of the first case. All staff and patients in this neurosurgical department were surveyed for any symptoms of fever (≥38°C) and enlarged tonsils during the outbreak period. Laboratory‐confirmed cases were defined as persons with positive HAdV55 PCR assays. Those not confirmed by the laboratory, but with symptoms and signs (fever (≥38°C) and enlarged tonsils), were defined as clinical‐confirmed cases (possible cases). Throat swabs from all cases were collected and tested. Information on demographic characteristics, onset of illness, and clinical symptoms were collected. For the use of all above‐mentioned specimens, written informed consent from all participants involved in the research was obtained (or from their parents or legal guardians in the case of a minor). This study was approved by the Ethics Committee of the Guangdong Provincial Center for Disease Control and Prevention, and was in compliance with the Helsinki Declaration.

### 2.2 Laboratory investigations

Throat swabs were kept and transported in a viral transport medium. Total viral nucleic acids (DNA and RNA) were extracted with QIAamp MiniElute Virus Spin Kits (Qiagen, Mississauga, Ontario, Canada) according to the manufacturer's instructions. For each specimen, assays for common respiratory viruses (respiratory syncytial virus, influenza virus A and B, parainfluenza virus 1‐3, human adenovirus, human enterovirus/rhinovirus, human metapneumovirus, human bocavirus, and human coronavirus) were performed with a one‐step reverse transcription‐polymerase chain reaction (qRT‐PCR). All primers are listed in Table 1. Viral isolation was performed for all collected throat swabs. Briefly, Hep‐2 cells incubated with swab specimens were cultured for three passages. The cytopathic effect (CPE) was checked. Cultures showing adenovirus‐like cytopathic effects were collected and analyzed with universal HAdV primers.

**Table 1:** RT‐qPCR primers and probes used in this study

Virus Tested	Sequences of Primers and Probes (5′‐3′)
Influenza viruses A	GAC CRA TCC TGT CAC CTC TGA C
	GGG CAT TYT GGA CAA AKC GTC TAC G
	FAM‐TGC AGT CCT CGC TCA CTG GGC ACG‐TAMRA
Influenza viruses B	TCCTCA ACTCACTCTTCG AGCG
	CGGTGCTCTTGACCAAATTGG
	FAM‐CCAATTCGAGCAGCTGAAACTGCGGTG‐TAMRA
Human parainfluenza‐1	CCTGATTTAAACCCGGTAATTTCTC
	TTCCTGCAGCTATTACAGAACATGAT
	FAM‐CCTATGACATCAACGACACA‐BHQ1
Human parainfluenza‐2	AGGACTATGAAAACCATTTACCTAAGTGA
	AAGCAAGTCTCAGTTCAGCTAGATCA
	FAM‐ATCAATCGCAAAAGCTGTTCAGTCACTGCTATAC‐BHQ1
Human parainfluenza‐3	TGATGGYTCAAACTCAACAACAAGAT
	CATACCCGAGAACTATTATTTTGCCTT
	FAM‐TATATCCCTGGTCCAACAGATG‐BHQ1
Human adenovirus	GGATGCTTCGGRGTACCTSAGT
	CCCCAKAYTGAAGTAGGTGTTCTGT
	FAM‐CCGGGTCTGGTGCAGTTCGCC‐BHQ1
Human metapneumovirus	CGTCAGCTTCAGTCAATTCAACAGA
	ATTARGTCCAADGATATTGCTGGTGTT
	FAM‐CTGCATTGTCTGAAAAYTGCCGCACAACATT‐BHQ1
Human rhinovirus/enterovirus	AGCCTGCGTGGCKGCC
	GAAACACGGACACCCAAAGTAGT
	FAM‐TCCTCCGGCCCCTGAATGYGGCTAA‐BHQ1
Human bocavirus	AGACGACGCCTAGTTGTTTGGT
	CAGTCCCTCCCAAGATACACTTTG
	FAM‐AGGTTCCACCCAATCCTGGTGCATTAAGC‐BHQ1
Human coronaviruses‐NL63	CATCAGGACCTTAAATTCAGACAACG
	GATTACGTTTGCGATTACCAAGACT
	FAM‐TAACAGTTTTAGCACCTTCCTTAGCAACCCAAACA‐BHQ1
Human coronaviruses‐229E	ATCAAAAGCTCCCAAATGGTGTT
	CTGTCACTTGAAGGATTCCGAGATT
	FAM‐CTGTTGTTGAAGAACCTGACTCCCGTGCTCC‐BHQ1
Human coronaviruses‐OC43	TAAGGGGTACTGGTACAGACACAACA
	ATGCGGTCCTGTTCCCAGATAG
	FAM‐CAGCCGATGGCAACCAGCGTCAACT‐BHQ1
Human coronaviruses‐ HKU1	CGACCAGGTTCACGTTCTCAAT
	GCAATCTCATCAGCCATATCAGGT
	FAM‐CACGTGGACCCAATAATCGTTCATTAAGTAGAAGTA‐BHQ1
Human respiratory syncytial virus	GCGTAACWACACCTKTAAGCACT
	CTTTGCTGYCTWACTATYTGAACATTG
	FAM‐ATCAATGATATGCCTATAACAAATGA‐BHQ1

### 2.3 Gene sequencing

The hexon, fiber, and penton genes of HAdV were amplified. The primers we used are listed as follows: Hexon‐F (5′‐CAGGATGCTTCGGAGT ACCTgAG‐3′) and Hexon‐R (5′‐ TTTCTGA AGTTCCACTCGTAGGTGTA‐3′); Fiber‐F (5′‐AGCGGCATACTTTCTCCATAC‐3′) and Fiber‐R (5′‐GGGAGGC AAAATAACTACTCG‐3′); and Penton‐F (5′‐ATCTG GTGGACAACAAGTCG‐3′) and Penton‐R (5′‐TCAGTAACGGTCACACGTTGG‐3′). The primers used to amplify the complete genome were designed according to HAdV55 reference viral genome sequences and are available upon request. The PCR products were sequenced with an ABI3730xl DNA Analyzer at IGE Biotech Co., Ltd. (Guangzhou, China). All sequences determined in this study were deposited in GenBank with accession numbers KY070248‐KY070273.

### 2.4 Phylogenetic tree

Multiple sequence alignments were performed with ClustalW,^15^ and alignments were manually edited with Aliview.^16^ For whole genome sequences, MAFFT software was used for alignment with default parameters (http://mafft.cbrc.jp/alignment/server/). Maximum likelihood (ML) trees were estimated in RaxML 17 using the generalized time‐reversible (GTR) nucleotide substitution model with gamma distribution among site rate heterogeneity.^18^

### 2.5 Statistical analysis

Data analysis was performed with spss 15.0 (SPSS Inc., Chicago, IL, USA). Differences with an error probability of *P*<.05 were regarded as significant. For categorical data, we used chi‐square testing and Fisher Exact testing as appropriate.

## 3 RESULTS

### 3.1 Outbreak epidemiology

**Figure 1:**
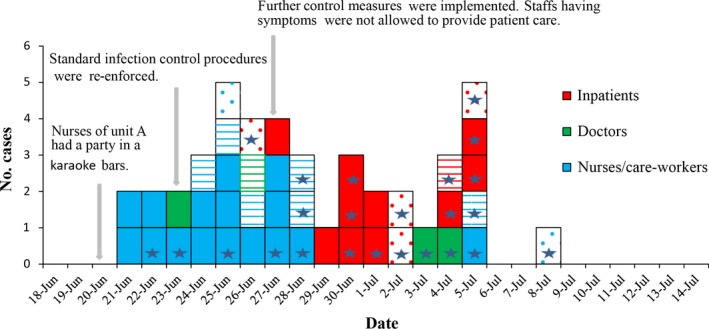
Case distribution during the adenovirus outbreak in a district hospital in Guangdong, China. Cases were identified on the basis of a case definition and an active search. Those full lines denote cases in unit A; horizontal lines denote cases in unit B; the dots denote cases in NSICU; the stars denote laboratory confirmed cases

The outbreak ranged from June 21 to July 8, 2016. The first cases were two nurses working at Neurosurgical Unit A. Our investigation identified 43 cases, including 24 laboratory‐confirmed cases and 19 possible cases. The onset of illness in all cases is shown in Figure 1. Two peaks were observed. The first one was from June 21‐28. It included 25 cases, among which 21 were nurses. The second was from June 29 to July 5. During this period, 13 of 15 infected inpatients were identified. It was observed that, within each unit, the first cases were all nurses. Table 2 illustrates the attack rate for each neurosurgical unit. The overall attack rate of this outbreak was 25.1%, with the highest attack rate of 46.8% in unit A, followed by 17.1% in the NSICU and 10.8% in unit B. Nurses had the highest attack rate. Twenty‐four of 66 nurses had developed similar respiratory symptoms during the outbreak, resulting in an attack rate of 36.4%. The attack rate for doctors and inpatients was 20.0% (3/15) and 16.7% (15/90), respectively.

**Table 2:** Case distribution during the adenovirus outbreak in a district hospital in Guangdong, China

Occupation	Number	Cases	Attack rate (%)
Unit A			
Nurses/care‐workers	21	16	76.2
Doctors^a^	7	2	28.6
Inpatients	33	10	30.3
Unit B			
Nurses/care‐workers	21	6	28.6
Doctors	7	1	14.3
Inpatients	46	1	2.2
NSICU			
Nurses/Care‐workers	24	2	8.3
Inpatients	11	4	36.4

a The acupuncturist responsible for the whole department is not included in this table.

All cases ranged from 1.5 to 72 years old, with 46.5% (20 cases) of cases in the range of 18‐19 years old. Detailed clinical information was collected (Table 3). Most cases (41 cases) reported upper respiratory symptoms. Fever was the most common symptom, followed by sore throat, cough, expectoration, headache, and runny nose. Twelve of these 43 cases developed pneumonia. No mechanical ventilation was needed. Two cases developed conjunctivitis. Most clinical symptoms and signs between infected inpatients and infected medical workers did not differ (Table 3). However, there were significantly more inpatients who developed pneumonia (*P*<.001).

**Table 3:** Clinical features of cases during the adenovirus outbreak in a district hospital in Guangdong, China

Symptoms	Total(n=43)	Inpatients (n=15)	Others (n=28)	*P* Value
Fever	37 (86.0)	12(80.0)	25(89.3)	0.65
Sore throat	28 (65.1)	5(33.3)	23(82.1)	0.00
Cough	17 (39.5)	6(40.0)	11(39.3)	0.96
Expectoration	11 (25.6)	3(20.0)	8(28.6)	0.72
Headache	11 (25.6)	0(0)	11(39.3)	0.01
Runny nose	8 (18.6)	1(6.7)	7(25.0)	0.23
Pneumonia	12 (27.9)	11(73.3)	1(3.6)	0.00
Conjunctivitis	2 (4.7)	0(0)	2(7.1)	0.54

Data are presented as No. (%)

### 3.2 Outbreak investigation and infection control measures

We reviewed all inpatients' medical records and surveyed their visitors. Neither the inpatients nor their visitors had respiratory symptoms before the outbreak. We also interviewed all the chief doctors and nurses working in these three units and enquired about the staff's routine practice in the involved neurosurgical department. There are separated doctor offices, nurse stations, and nurse resting rooms within each unit. The staff had basically followed the standard infection control procedures when they cared for the patients. In the nurse stations and the resting rooms, the nurses preferred not to wear any protective equipment. They shared most office supplies, such as computers, medical records, and examination records. More often, it was the nurses who visited the doctors' offices for daily work communication, while the doctors rarely visited the nurse stations and their resting rooms, except for the acupuncturist, who was responsible for the entire department and was used to drinking water in nurse station A. There was a dispensing counter in nurse station A from which nurses from unit B and the NSICU sometimes pick up medicines. Other interactions between units were fewer. One day before the outbreak, nurses from unit A had a party in a karaoke bar.

Control measures were implemented from June 23, when the hospital infection control committee was informed of this possible nosocomial infection. Environmental cleaning was enhanced. Self‐protections, including hand hygiene and surgical mask wearing, were re‐enforced. All cases and their close contacts were followed up. On June 27, due to the increasing number of cases, further control measures were implemented. Staff with symptoms were either given medical leave or treated in isolation, as what had been implemented for the infected inpatients. The personnel entering and leaving the involved neurosurgical department were restricted. Visitors to the department were required to wear surgical masks. In addition, screenings for similar symptoms were performed in the entire hospital. Specimens from cases and the environment were collected and sent to the local CDC for pathogen detection.

### 3.3 Laboratory results

Viral testing was performed from June 28 to July 14 for 36 cases (Samples from the other seven cases were not available). A total of 64 respiratory samples were collected; 27 specimens from 24 patients were HAdV‐positive. No other respiratory virus was identified. In addition, 29 serum samples, 13 fecal samples, and one urine sample were tested, among which six, eight, and one were HAdV‐positive, respectively. HAdVs were successfully isolated for eight patients. To determine the viral type, hexon, fiber, and penton genes were amplified. These sequences were blasted in GenBank. All genes exhibited 100% similarity with HAdV55 strain HAdV55 XZ2012‐492 (accession number KC857701). Furthermore, 37 environmental samples from the department and personnel, such as stethoscopes, doorknobs, office computers, and medical records, were also collected for viral testing, none of which showed positive results.

### 3.4 Phylogenetic analysis

**Figure 2:**
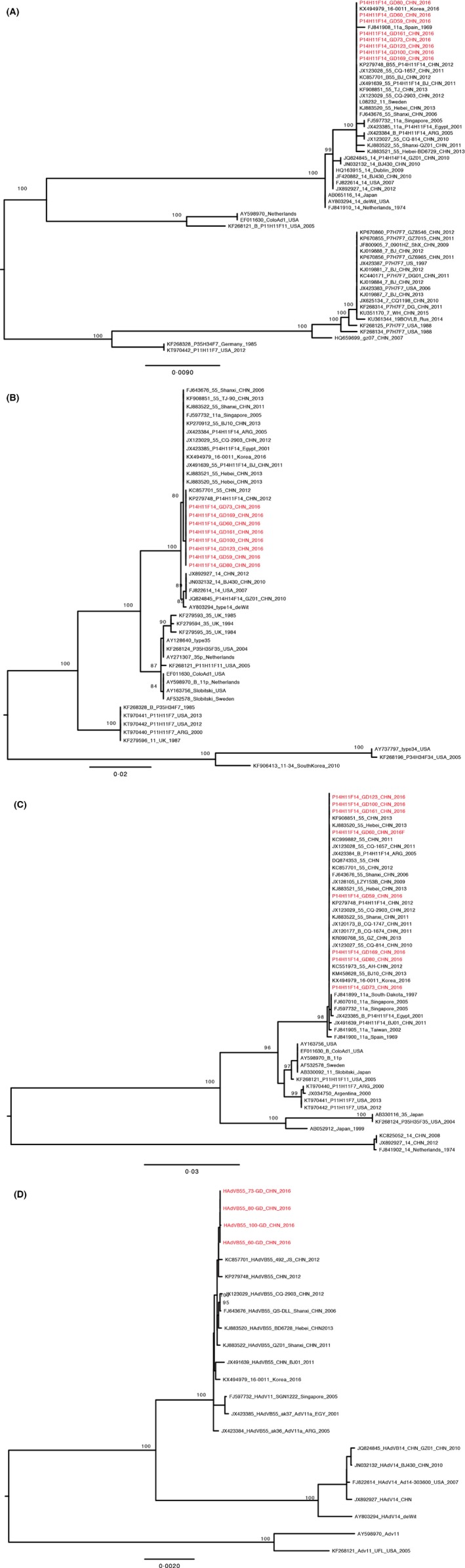
Phylogenetic analysis based on the fiber (A), penton (B), hexon (C), and the whole genome sequence (D). Phylogenetic analysis is conducted using the maximum likelihood method and 1000 bootstrap replicates using the RaxML program. HAdV isolates from this outbreak are indicated by red color. Reference strains are labeled with their GenBank accession numbers, countries of collection, and dates of collection (if available). Branches with supporting bootstrap value over 80% are indicated

The fiber gene (1048 nucleotides (nt)), hexon gene (1603 nt), and penton gene (1206 nt) were obtained from eight HAdV strains isolated in the outbreak. Sequences of each gene were completely identical, suggesting the outbreak was caused by the single viral introduction. Phylogenetic trees of fiber, hexon, and penton genes were constructed by integrating all closely related adenovirus sequences from GenBank (genetic similarity>80%, coverage>90%). The phylogenies showed the outbreak strains belonged to the [P14H11F14] genotype, with hexon genes clustered together with HAdV‐B11, and the fiber and penton genes clustered with HAdV‐B14 (Figure 2). This genotype is a recently emerged recombination between HAdV‐B11 and HAdV‐B14 viruses named HAdV55. To confirm the type of HAdV, four randomly selected HAdV strains from two cases with upper respiratory infections and two cases with pneumonia were sequenced for the whole genome (accession number KY070248, KY780931‐ KY780933). No variations were observed. The phylogeny of the whole genome sequence confirmed that the outbreak strains fell into the HAdV55 [P14H11F14] clade (Figure 2D). Strains from this outbreak were segregated together and could be separated from other isolates. The most closely related strains were HAdV55 strains that previously circulated in China between 2006 and 2013.

## 4 DISCUSSION

This study identified a cluster of adenovirus type B55 infection in the neurosurgical inpatient department of a general hospital. Through targeted surveys, the possibility of inpatients as the source of this outbreak was excluded. We suggested that the pathogen of this outbreak may have been introduced into the hospital by nurses in unit A. They had a party in a karaoke bar 1 day before the outbreak. One or some of them may have introduced the virus from the bar into unit A and then spread it to other units. Other possibilities may exist, as well. One of the first two cases had been reported a 2‐day leave 6 days before she was infected. She may have been infected from her community and then introduced the virus to the hospital. The party offered a good chance for viral transmission among the nurses. Transmission from hospital staff to inpatients may have occurred during this outbreak. The interval between the two peaks of the outbreak was about 6 days, approximately equal to the incubation period of the HAdV infection.^19^ The later peak mainly consisted of inpatients, which may represent secondary cases. Molecular analysis revealed 100% identities of the fiber, hexon, and penton genes from HAdVs isolated in this outbreak. All fell into HAdV55 [P14H11F14] cluster, indicating that HAdV55 was the single viral strain for the outbreak.

HAdV55 is a newly identified re‐emerged acute respiratory disease pathogen. When and where this new lineage arises is still unknown due to the lack of surveillance. Current sequences data indicate the oldest strain of HAdV55 [P14H11F14] was identified in Egypt in 2001. In recent years, this virus has been reported to have caused several outbreaks, the majority of which arose in military training facilities. In China, HAdV55 was first identified in 2006 from an outbreak in a senior high school in the Shaanxi province.^9^ Since then, sporadic cases of HAdV55 infections have been increasingly reported, especially in northern China.^14^, ^20^ This first identification of HAdV55 outbreak in southern China suggests this recombinant adenovirus has been widely disseminated in China. The limited data suggest the outbreak strains of HAdV55 in Guangdong were more likely introduced from other regions of China rather than from recombination in local places, as nearly identical strains were identified from Beijing and Chongqing cities for these three genes. Comparative studies conducted between HAdV55 and other types (HAdV‐7, HAdV‐3, HAdV‐14, HAdV‐50, and HAdV‐C) show a higher tendency of more severe illness in HAdV55‐infected patients.^20^ In this outbreak, 11 of 15 infected inpatients developed pneumonia. In contrast, only one staff member was diagnosed with pneumonia, and other staff developed mild and self‐limited symptoms. One possible reason may be body immune competence. Recent studies have shown that the immune system plays a crucial role in the clearance of HAdV viremia and survival of the host.^21^, ^22^ Owing to underlying conditions, it is more likely for the infected inpatients to develop severe diseases.^23^, ^24^ Given the possible association of severe disease with HAdV‐55, this virus should be considered sooner in such hospital settings to allow appropriate medical care.

Healthcare‐associated infection has been a constant issue since the early days of medicine. To our knowledge, this is the first report on an HAdV55‐associated hospital outbreak. HAdV is an ideal agent for nosocomial transmission due to its prolonged shedding and its resilient nature. It can spread rapidly through close contact with infected patients or by exposure to fomites. During this outbreak, standard infection control procedures, such as hand washing, wearing surgical masks, and hair caps, were implemented as routine policy in the hospital. However, some staff may have adopted these infection control precautions improperly due to negligence or weak control awareness. Furthermore, consistent compliance with contact and droplet precautions only occurred in working areas, but not in the nurse stations and resting rooms during the beginning of the outbreak. The fact that the infected cases remained infectious during the incubation period has generated challenges to infection control. It was not possible to assess the degree of contact between the first two cases and the other infected staff and inpatients. The acupuncturist and some infected nurses in other units had reported visiting histories to unit A before their illness. Restriction for staff to perform patient care was implemented on June 27. It is possible that this measure helped prevent further transmissions from nurses to patients, as no additional inpatients were infected after July 5, about one incubation period past its implementation. To combat nosocomial outbreaks, healthcare settings should ensure that universal precautions and healthcare guidelines are consistently followed.

Our study has limitations. First, several respiratory viruses could cause respiratory infections.^25^ Previously published data show that it is difficult to make a reliable distinction among these pathogens based on clinical signs and symptoms.^26^ To ensure case findings, we used a sensitive definition of possible cases in this outbreak, which may include both adenovirus and non‐adenovirus infections, as well as infected patients who may not have been involved in this infection cycle. Our investigation found 27 throat swab specimens with a HAdV‐positive status, while pathogens for the remaining cases were not fully clarified. Therefore, the attack rate may have been overestimated. More laboratory tests should be carried out to understand pathogenic characteristics. Second, based on our investigation, we implicated that the virus could be intruded from outside of the hospital by the nurse or nurses in unit A. However, without environmental samples and surveys from the bar and the aforementioned nurse's community, the transmission chain cannot be fully clarified. Other possibilities cannot be excluded. Recently, an increasing frequency of HAdV‐55 infection was observed among patients with acute respiratory disease. However, we have little knowledge on HAdV‐55 distributions and their molecular evolution in southern China at present. The first HAdV‐55 identified in Guangzhou was in 2013. A more comprehensive investigation combined with sequential surveillance and monitoring of this agent would be helpful to trace the transmission origin and provide more molecular epidemiology baseline data in China.

## DECLARATION OF INTEREST

The authors declare no conflict of interest.

## AUTHORS 'CONTRIBUTIONS

JW and CK planned and designed the study. LY, LZ, and JL took part in drafting the manuscript. JL and LY conducted the sequence analysis. Experiments were performed by LZ, LY, YS, JS, XZ, LL, and HN. Clinical data and patient samples were provided by JW, CK, and MK. All authors read and approved the final manuscript.
